# Processing of the major autolysin of *E*. *faecalis*, AtlA, by the zinc-metalloprotease, GelE, impacts AtlA septal localization and cell separation

**DOI:** 10.1371/journal.pone.0186706

**Published:** 2017-10-19

**Authors:** Emily K. Stinemetz, Peng Gao, Kenneth L. Pinkston, Maria Camila Montealegre, Barbara E. Murray, Barrett R. Harvey

**Affiliations:** 1 Center for Molecular Imaging, Brown Foundation Institute of Molecular Medicine for the Prevention of Human Diseases, University of Texas Health Science Center, Houston, Texas, United States of America; 2 Department of Microbiology and Molecular Genetics, University of Texas Health Science Center, Houston, Texas, United States of America; 3 Division of Infectious Disease, Department of Internal Medicine, University of Texas Health Science Center, Houston, Texas, United States of America; Centre National de la Recherche Scientifique, Aix-Marseille Université, FRANCE

## Abstract

AtlA is the major peptidoglycan hydrolase of *Enterococcus faecalis* involved in cell division and cellular autolysis. The secreted zinc metalloprotease, gelatinase (GelE), has been identified as an important regulator of cellular function through post-translational modification of protein substrates. AtlA is a known target of GelE, and their interplay has been proposed to regulate AtlA function. To study the protease-mediated post-translational modification of AtlA, monoclonal antibodies were developed as research tools. Flow cytometry and Western blot analysis suggests that in the presence of GelE, surface-bound AtlA exists primarily as a N-terminally truncated form whereas in the absence of GelE, the N-terminal domain of AtlA is retained. We identified the primary GelE cleavage site occurring near the transition between the T/E rich Domain I and catalytic region, Domain II via N-terminal sequencing. Truncation of AtlA had no effect on the peptidoglycan hydrolysis activity of AtlA. However, we observed that N-terminal cleavage was required for efficient AtlA-mediated cell division while unprocessed AtlA was unable to resolve dividing cells into individual units. Furthermore, we observed that the processed AtlA has the propensity to localize to the cell septum on wild-type cells whereas unprocessed AtlA in the Δ*gelE* strain were dispersed over the cell surface. Combined, these results suggest that AtlA septum localization and subsequent cell separation can be modulated by a single GelE-mediated N-terminal cleavage event, providing new insights into the post-translation modification of AtlA and the mechanisms governing chaining and cell separation.

## Introduction

*Enterococcus faecalis*, a Gram-positive commensal bacterium of the gastrointestinal tract, is an important pathogen in hospital-acquired infections (HAIs) [1,2]. *E*. *faecalis* is capable of establishing surface communities known as biofilms on both human tissue and medical devices [3–5]. Due to increasing numbers of antibiotic resistant isolates and difficulty in eradicating biofilms, enterococcal infections have become a significant challenge in healthcare [6,7]. Thus, there is increased urgency to improve our understanding of the underlying factors that contribute to *E*. *faecalis* virulence in order to pursue alternative approaches in medical treatments.

AtlA, an autolysin involved in peptidoglycan hydrolysis [8], plays an important role in the separation of daughter cells following replication. In *E*. *faecalis*, an *atlA* deletion mutant presents a long chaining phenotype under light microscopy [8], with strings of cells attached end to end due to incomplete septum cleavage. The impact of AtlA on septal cleavage is further demonstrated by the addition of AtlA protein to an *atlA* deletion mutant, resulting in short chaining cells [9]. Evidence suggests that conditions promoting chain formation may promote virulence in Gram-positive bacteria by encouraging adherence and colonization in the host, given that longer forms would have more adhesins available per particle and exhibit improved avidity due to increased protein-ligand interactions [10,11]. In addition, evidence suggests that AtlA is a major contributor to biofilm formation through its autolytic activity as an *atlA* deletion strain demonstrated minimal autolysis using standard autolytic assays and are attenuated in their ability to form biofilms [8,12]. Furthermore, it was recently demonstrated that AtlA control over chain length greatly impacts the virulence of *E*. *faecalis* in a zebrafish model of infection. [13].

An extracellular zinc metalloprotease known as gelatinase (GelE), which is regulated by the Fsr-quorum sensing system of *E*. *faecalis*, has been identified as a major virulence factor in the establishment of infection through bacterial adherence and biofilm formation [12,14–18]. GelEs contribution to virulence is partially attributed to the role it plays in the regulation of AtlA. A mechanism has been previously proposed by which proteolytic processing of AtlA mediates lysis of bacterial cells, which in turn releases extracellular DNA (eDNA), thus stabilizing biofilm [19,20]. Consistent with evidence demonstrating a role for GelE in the regulation of AtlA activity, Δ*gelE* mutants exhibit attenuated autolytic ability and also demonstrate a chaining phenotype as observed by microscopy, albeit less pronounced than *atlA* mutants [21].

Through sequence comparison to other autolysins, it was determined that AtlA is composed of three domains [22]. Domain I has been defined as a T/E rich domain with no known function; Domain II contains the enzymatic activity region responsible for peptidoglycan hydrolysis; while Domain III contains six LysM residues necessary for anchoring AtlA to the cell wall through recognition of N-acetylglucosamine (GlcNAc) residues of peptidoglycan [23]. Eckert *et al*. demonstrated that, while the C-terminal region was necessary for enzymatic activity, this was likely a consequence of orienting Domain II more proximal to its peptidoglycan cell wall substrate [22].

In this study, monoclonal antibodies were developed which recognize different domains of AtlA as tools to improve our understanding of the impact protease-mediated post-translational modification of AtlA has on cellular function. Using N*-*terminal sequencing, a GelE cleavage site on AtlA was identified which removed the T/E rich N-terminus of the protein. Our analysis demonstrated that loss of the N-terminus did not affect the ability of AtlA to hydrolyze peptidoglycan, but rather directly affected the localization of surface associated AtlA to the cell septum, and its ability to separate daughter cells. Furthermore, we discuss the potential implications that post-translation modification of AtlA could have on *E*. *faecalis* virulence by regulating colonization and dispersion of infection.

## Materials and methods

### Chemicals

Unless otherwise indicated, all culture media were purchased from Difco Laboratories and all chemicals were purchased from Sigma (St. Louis, MO). Brain heart infusion (BHI) and Luria broth (LB) were prepared as described by the manufacturer (Becton, Dickinson). Bacto agar was used as a solidifying agent for all semi-solid media. Oligonucleotides were purchased from Sigma (St. Louis, MO).

### Bacterial strains and culture conditions

All *Enterococcus* strains were grown in BHI broth or on BHI agar plates at 37°C. *E*. *coli* strains used for protein purification were grown in LB at 37°C. The *E*. *faecalis* strains used included OG1RF [24], TX5264 (OG1RF Δ*gelE*) [25], JH2-2 [26], TX5127 (OG1RF Δ*atlA*) [8], TX5243 (OG1RF *sprE* insertion mutant [*▽sprE*]) [17], TX5471(OG1RF Δ*gelE*Δ*sprE*) [27], and OG1RF Δ*atlA*Δ*gelE* (developed in this study). If required, growth medium was supplemented with antibiotics at the following concentration: 100 μg/mL ampicillin, 200 μg/mL gentamicin, 100 μg/mL rifampicin, 25 μg/mL fusidic acid, and 2000 μg/mL kanamycin.

### Construction of the *atlA* and *gelE* deletion mutant

Deletions were generated as previously described [28]. The primer pairs AtlAUpFor (5’- CGGGATCCTGACTGATTTTTCCGCTTG -3’)/ AtlAUpRev (5- CATTGCCACGTGACATTGATTCTTTCTTC -3’) and AtlADownFor (5’- CAATGTCACGTGGCAATGCTTCTTCAAC -3’)/ AtlADownRev (5’- CGGAATTCGCTCCTTCAGAACGTCCA -3’) were used to amplify the sequences upstream and downstream of the *atlA* gene. The forward primer of the upstream region was designed to incorporate a BamHI site, and the reverse primer of the downstream region was designed to incorporate an EcoRI site. These two amplicons were joined by PCR overlap extension[29], resulting in a 5’ BamHI site and a 3’EcoRI site which were used for insertion in the recipient pHOU1 vector [30]. The resulting plasmid was then introduced into *E*. *faecalis* CK111 by electroporation and then filter mated with *E*. *faecalis ΔgelE* (TX5264). The *ΔatlAΔgelE* double deletion mutant was selected by culturing the colonies on minimal medium 9-yeast extract-glucose (MM9YEG) plates supplemented with 10 mM p-chloro-phenylalanine, and verified using two sets of primers outside the deleted region and pulsed field gel electrophoresis (PFGE) was used to verify the correct background.

### Recombinant protein construction, expression, and purification

Expression and purification of rAtlA was carried out as previously described[22,31]. All recombinant proteins were constructed and purified in a similar manner using different primers for amplification. A DNA fragment encoding AtlA was amplified from OG1RF by PCR with the primers AtlABamHIFor (5’-GCAGGATCCACAGAAGAGCAGCCAACAAATGC-3’) and AtlAKpnIRev (5’-CTCCGGTACCTCATTAACCAACTTTTAAAGTTTGACC-3’) and ligated into pQE30 (Qiagen, Inc.) using BamHI and KpnI sites. The resulting plasmid was transformed into M15 *E*. *coli* harboring pREP4 (Qiagen, Inc.). Recombinant AtlA (rAtlA) begins with the amino acids TEEQPT and ends with the amino acids QTLKVG. A DNA fragment encoding AtlA D2D3 (Domain 2-Domain 3 truncated AtlA) was amplified from OG1RF by PCR with the primers AtlAD2BamHIFor (5’-GCAGGATCCGAATTTATTGCCGAGTTAGCTCG-3’) and AtlAKpnIRev (above). Recombinant D2D3 (rD2D3) begins with the amino acids SEFIAE and ends with the amino acids QTLKVG. A DNA fragment encoding AtlA D3 (Domain 3 truncated AtlA) was amplified from OG1RF by PCR with the primers AtlAD3BamHIFor (5’- GCAGGATCCCCATCTTCTGGT- 3’) and AtlAKpnIRev (above). Recombinant D3 (rD3) begins with amino acids TPSSGGNT and ends with the amino acids QTLKVG. A DNA fragment encoding AtlA’ (N-terminally truncated at identified GelE cleavage site) was amplified from OG1RF by PCR with the primers AtlA’BamHIFor (5’-GCAGGATCCTTATCACCGACGCAAAGTCC-3’) and AtlAKpnIRev (above). Recombinant AtlA’ (rAtlA’) begins with the amino acids LSPTQ and ends with the amino acids QTLKVG.

### Development of anti-AtlA mAbs

Monoclonal antibody (mAb) generation against recombinant AtlA (rAtlA) was carried out as previously described [27,32]. Briefly, 6–8 week old BALB/c (Harlan) mice were immunized in three subcutaneous sites with 10–20μg rAtlA protein emulsified into Freunds Complete Adjuvant (CFA, SIGMA) followed by 2 subsequent immunizations via intraperitoneal injection with protein emulsified into Freunds Incomplete Adjuvant (IFA, SIGMA) at 10–14 day intervals. Following the third immunization, serum titers were determined via ELISA against rAtlA. One mouse was selected for final intraperitoneal boost in Phosphate Buffered Saline (PBS). Mouse splenocytes were collected 3 days post injection and fused with SP2/0 mouse myeloma cells [33]. MAbs from parental wells were evaluated for binding specificity to rAtlA and native surface displayed antigen on OG1RF cells via ELISA and whole-cell ELISA [34,35], followed by kinetic screening for the highest affinity clones using surface plasmon resonance (SPR) on a Biacore T100 (GE Healthcare) as previously described [36].

### Specificity ELISA

Specificity ELISA was carried out as previously described [32] with slight modifications. Briefly, Microlon 600 ELISA microtiter plates (Greiner Bio-one, NC) were coated with 10 μg/mL of recombinant proteins (rAtlA, rD2D3, and rD3) in 50 mM carbonate buffer at 4°C overnight. Plates were washed 3 times with PBS-0.2% Tween 20 (PBST) and then incubated with 5% dry milk in PBST to block nonspecific binding. Anti-AtlA mAbs at 10 μg/ml were used for primary binding for 1 hr at RT followed by a wash and the addition of 100 μl of a 1:3,000 dilution of goat anti-mouse IgG-HRP in 5% milk-PBST (Jackson Immunoresearch). After 1 h of incubation at RT, the wells were washed three times with PBS–0.2% Tween 20. Tetramethylbenzidine (TMB) substrate was added, followed by incubation at room temperature for 10 min, before stopping the reaction with the addition of 2M H_2_SO_4_. The absorbance of each well was measured at an optical density at 450 nm (OD_450_).

### Flow cytometry analysis

Flow cytometry analysis was performed as previously described [32]. *E*. *faecalis* cells in BHI and appropriate antibiotics were harvested at an OD_600_ equivalent of 0.4, washed twice with 2% BSA-PBS and labeled for 0.5 hour at 25°C with 10 μg/mL of either anti-AtlA mAb 44 or 88 in 2% BSA-PBS. After two washes, the cells were labeled with 1:100 R-phycoerythrin (PE) conjugated goat F(ab’)_2_ anti-mouse IgG (Fc) (Jackson Immunoresearch) in 2% BSA-PBS for 0.5 hour at 25°C in the dark. The cells were then washed, followed by fixation with 1 ml of 1% paraformaldehyde for analysis on a Becton Dickenson FACSCalibur flow cytometer (BD Bioscience, CA). For each sample, 10,000 gated events were collected for analysis.

### Western blotting

*E*. *faecalis* cells were harvested after 4 hours of growth at 37°C. Cell pellets were resuspended in SDS-PAGE sample buffer, and heated for 10 minutes at 95°C. The samples were loaded on a 10% Tris-glycine gel under reducing conditions in MOPS (morpholinepropanesulfonic acid buffer) and transferred to an Immobilon-P polyvinylidene difluoride (PVDF) membrane (Millipore) according to manufacturer’s protocol. Membranes were probed with anti-AtlA mAbs 44 or 88 at 10 μ/mL followed by HRP-conjugated goat anti-mouse IgG secondary antibody and developed using the TMB substrate system (KPL).

### Peptidoglycan hydrolase activity assay

To purify peptidoglycan free of AtlA [22,37], OG1RF Δ*atlA* cells were grown to an OD_650_ of 0.7. The bacteria were centrifuged and peptidoglycan was extracted by treating the pellet with 14 mL 4% sodium dodecyl sulfate (SDS) for 30 minutes at 100°C. Peptidoglycan was washed five times with 20 mL of water and was then incubated with Pronase (200 μg/mL) in 1 mL of Tris-HCl (10 mM) and with trypsin (200 μg/mL) in 1 mL of phosphate buffer overnight at 37°C. Peptidoglycan was washed two times with 20 mL of water. To digest peptidoglycan with recombinant protein, 10 μg/mL of either recombinant AtlA or AtlA’ was added to purified peptidoglycan. Turbidity at 450 nm was measured every 30 minutes on a Genesys 20 Spectrophotometer (Thermo Fisher) over a 6-hour time period at 37°C in 25 mM Tris-HCl pH 7.5, 100 mM NaCl.

### N-terminal sequencing

For rAtlA cleavage, reaction mixtures containing 10 μg of rAtlA and 10 μg/mL purified GelE in 0.1 mL PBS were incubated for 30 minutes at 37°C. The enzyme activity was stopped by the addition of SDS-PAGE sample buffer containing 2-mercaptoethanol followed by boiling. Samples were applied onto SDS-PAGE gradient gels (4 to 20%) (NuSep, Inc.), followed by a transfer to an Immobilon-P polyvinylidene difluoride (PVDF) membrane (Milipore) according to manufacturer’s protocol. The band representing cleaved AtlA was cut from the PVDF membrane and the Protein Chemistry Laboratory at Texas A&M University (College Station, TX) was contracted to complete the N-terminal sequencing.

### Gelatinase-AtlA cleavage assay

Cleavage assays used purified GelE. Purified GelE was obtained from *▽sprE* as described previously [27,38,39]. Briefly, cells were grow to stationary phase and the culture medium was filtered with 0.45 and 0.22 μm pore-size filters. Proteins were precipitated overnight at 4°C with the addition of ammonium sulfate crystals to a saturation of 60%. After centrifugation, the protein pellet was dialyzed overnight 4°C in 50 mM Tris-HCl-1 mM CaCl_2_, pH 7.8. A second concentration step was carried out using a Millipore stirred-cell concentrator with a 10,000-molecular weight cutoff membrane. Sample was then applied to a HiPRep 16/60 Sephacryl S-200 high-resolution column (GE Healthcare) and tested for gelatinase activity via gelatin agar plates [40].

For rAtlA cleavage, reaction mixtures containing 3 μg of rAtlA and various amounts of purified GelE (ranging from 0 to 1.5 μg) in 0.1 ml of PBS were incubated for 30 minutes at 37°C. The enzyme activity was halted by the addition of SDS-PAGE sample buffer containing 2-mercaptoethanol followed by boiling. Purified GelE (1.5 μg) and rAtlA (3.0 μg) were diluted in 0.1 ml of PBS and incubated similarly to serve as controls. Samples were applied onto SDS-PAGE gradient gels (4 to 20%) (NuSep, Inc.), and developed with Coomassie blue protein stain (Sigma).

### Light microscopy

Chaining phenotype was determined as previously described with slight modifications [41]. *Enterococcus* cells were grown overnight in BHI medium with 100 μg/mL rifampicin and 25 μg/mL fusidic acid. The following morning the cells were diluted to an OD_600_ of 0.1 and were grown at 37°C for 3.5 hours while shaking at 240 RPM. 5μg/mL recombinant full-length AtlA or cleaved AtlA (AtlA’) was then added to either the Δ*atlA* or Δ*atlA*Δ*gelE* strains and allowed to incubate at 37°C while shaking for 0.5 hour. As controls, OG1RF, Δ*atlA* and Δ*atlA*Δ*gelE* strains were incubated in parallel without the addition of recombinant proteins. Cells were then harvested and resuspended in 8 mL 0.9% saline. Finally, 10 μL was spotted on a glass slide, covered with a coverslip and visualized under a light microscope at 1000x magnification. The numbers of cells per chain were recorded for 50 randomly chosen chains of each strain and classified for length based on criteria defined in previous work [41].

### Immunogold electron microscopy

Transmission electron microscopy (TEM) experiments were carried out as previously described [42]. Briefly, *E*. *faecalis* cells grown overnight in BHI medium were centrifuged and washed once with 0.1 M NaCl and twice with PBS. Next, 10 μL of bacterial suspension in PBS was incubated on a nickel-coated carbon grid for 1 minute and the samples were washed three times with 1% BSA in PBS. Samples were blocked for an hour at 25°C with 0.1% gelatin in PBS. Following a 1% BSA-PBS wash, the samples were labeled with α-AtlA mAb 44 (10 μg/mL in 1% BSA-PBS) for 1 hour at 25°C. The samples were then washed with 1% BSA-PBS three times and blocked with 0.1% Gelatin in PBS for 1 hour at 25°C. Following the second blocking step, the samples were labeled with 1:20 diluted 18 nm affinipure gold-conjugated anti-mouse IgG (Jackson ImmunoResearch, Lot #131575) in 1% BSA-PBS for 1 hour at 25°C. Subsequently, the samples were stained with 0.1% uranyl acetate in H_2_O for 30 seconds and imaged on a JEOL JEM-1400 electron microscope.

Quantification analysis of AtlA localization was done using TEM images as previously described [43]. Forty representative bacteria cells at the late phase of separation with clear septa were analyzed from each OG1RF and Δ*gelE* strain. Each bacterium was divided into 3 equal regions from the center septum to pole. Numbers of gold particles in each region were counted and comparison was made between the two strains.

## Results

### Anti-AtlA mAbs display distinct AtlA binding profiles

**Fig 1:**
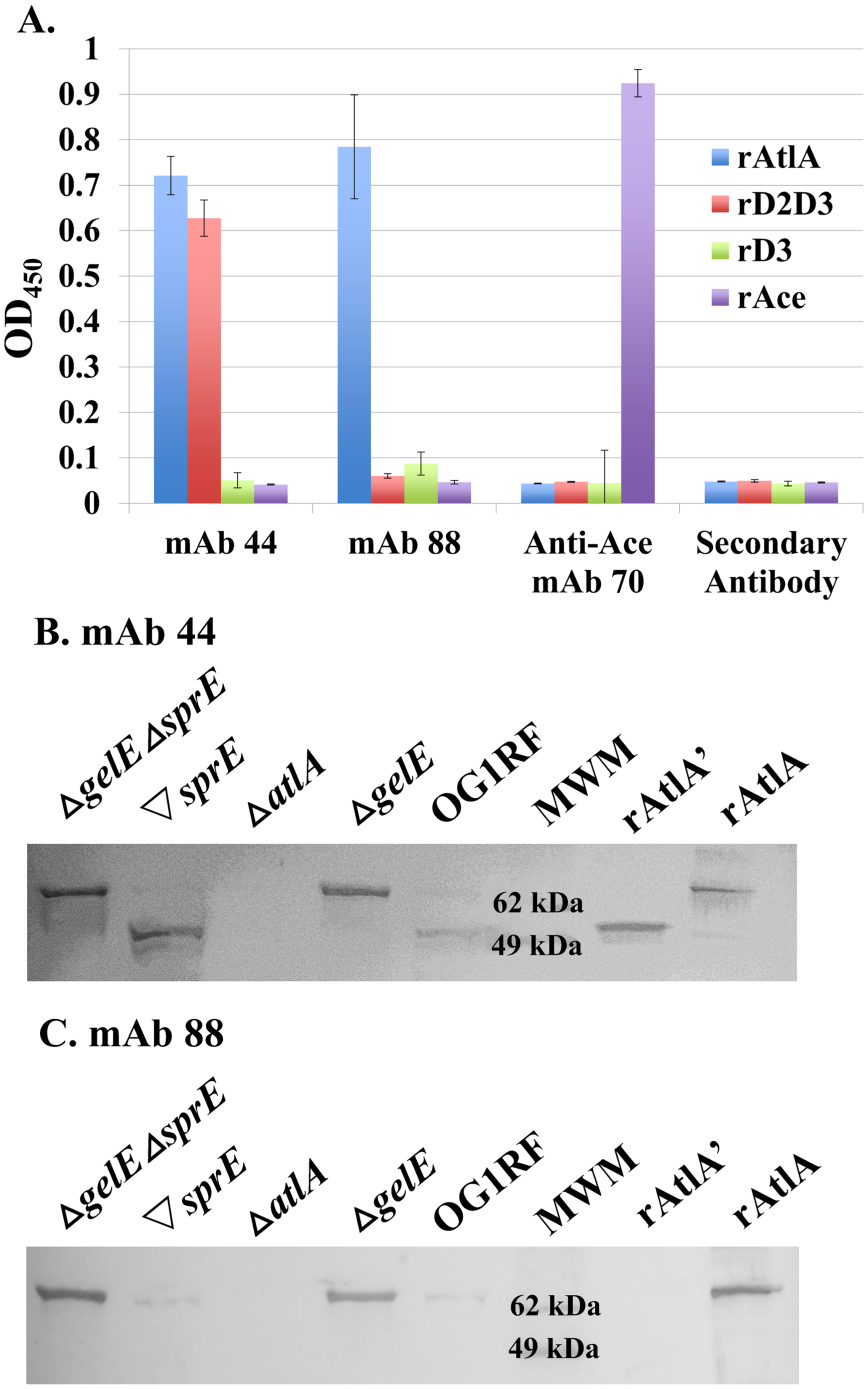
Anti-AtlA mAbs 44 and 88 distinguish GelE-truncated AtlA from full-length AtlA. (A) Anti-AtlA mAb 44 and 88 were evaluated via ELISA for their ability to bind to recombinant full-length AtlA, D2D3, and D3. Anti-Ace mAb 70 and secondary antibody alone were run as controls. Results shown are averages of three independent experiments and error bars represent 1 standard deviation of the mean. (B and C) Supernatants collected from whole cell lysates of OG1RF, *ΔgelE*, *ΔatlA*, *ΔsprE*, and *ΔsprE ΔgelE* strains were probed in Western blots with either anti-AtlA mAb 44 (B) or mAb 88 (C) as the primary antibody. Results shown are representative of three independent experiments.

It was previously discovered that GelE cleaves AtlA. However, GelE cleavage sites in AtlA were not reported [20]. To further investigate the protease-mediated cleavage of AtlA, we developed in house two high affinity mouse anti-AtlA monoclonal antibodies (mAb44 and mAb 88) and analyzed their binding profiles to various AtlA domain constructs using ELISA. As shown in Fig 1A, mAb 44 and 88 demonstrate different binding profiles against recombinant full-length AtlA (rAtlA), AtlA Domain II, III (D2D3), and AtlA Domain III (D3), suggesting that anti-AtlA mAb 88 recognizes only full-length rAtlA, mAb 44 recognizes both the full-length rAtlA and rD2D3, while neither mAb recognized rD3 AtlA. A recombinant version of the *E*. *faecalis* surface protein, Ace, and an Ace specific antibody mAb 70 were used as irrelevant protein and irrelevant mAb controls, respectively. These results indicate that mAb 44 and 88 bind to distinct binding epitopes on AtlA and might be used to distinguish AtlA cleavage events.

Using the domain-specific antibodies 44 and 88, we carried out Western blot analysis to evaluate AtlA extracted from wild-type OG1RF versus Δ*gelE* grown to stationary phase. Additionally, we included strains incapable of producing SprE, (*▽sprE*, and Δ*gelE*Δ*sprE*) a serine protease co-transcribed with GelE, which has previously been reported to process AtlA [20]. When mAb 88 was used as the primary antibody, a single band comparable in size to that of recombinant full length AtlA (molecular weight 71.4 kDa) was detected in *gelE* deletion strains (Δ*gelE* and Δ*gelE*Δ*sprE*), but not detected in the GelE producing strains (OG1RF and *▽sprE*) (Fig 1B). When detecting with mAb 44 however, strains that produce GelE show a distinct band smaller in size than that of recombinant full length AtlA, suggesting cleavage of AtlA by GelE (Fig 1B). Together with ELISA results in Fig 1A suggesting that mAb 88 binds within the N-terminus of AtlA, we propose that AtlA cleavage occurs in *E*. *faecalis* in presence of GelE, and that cleavage removes an N-terminal sequence of the protein.

### Surface bound AtlA is present as a truncated form when in the presence of GelE

**Fig 2:**
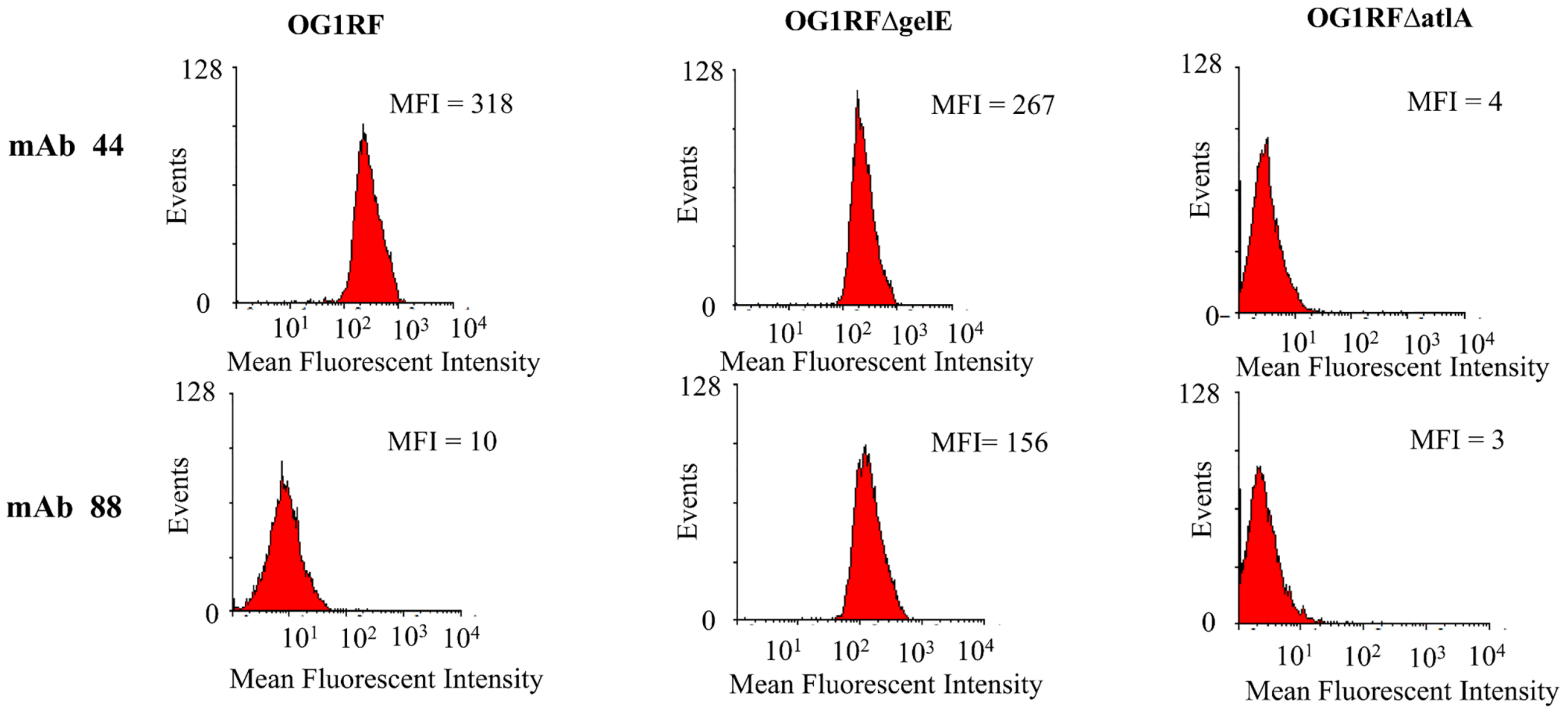
A surface-associated truncated form of AtlA is present in the presence of GelE. Flow cytometry analysis of wild-type OG1RF, *ΔgelE*, and *ΔatlA* strains with either anti-AtlA mAb 44 or mAb 88 demonstrate two unique binding profiles. mAb 88 recognized AtlA on the surface in the *ΔgelE* strain while mAb 44 recognized AtlA in both the wild-type OG1RF and the *ΔgelE* strain. The *ΔatlA* strain was used as a control to demonstrate specificity of mAb binding. Results shown are representative of three independent experiments.

Consistent with the ELISA and Western blot results, two distinct binding profiles became apparent while assessing the ability of anti-AtlA mAb panel to bind to surface bound AtlA on both exponentially grown wild-type *E*. *faecalis* (OG1RF) and Δ*gelE* cells using flow cytometry. Evaluation at exponential phase assured that Fsr mediated GelE expression would be induced in the wild-type [27]. The mAb 88 demonstrated specific binding to the Δ*gelE* strain surface with a MFI of 156, compared to a MFI of 10 for the OG1RF. In comparison, mAb 44 bound well to both the wild-type OG1RF and the Δ*gelE* strain, with MFIs of 318 and 267, respectively, (Fig 2). Δ*atlA* was used as a negative control to demonstrate that the antibodies utilized showed minimal cell binding in the absence of AtlA (MFI of single digits). These results, together with Western blot analysis, suggest that a GelE processed truncated form of AtlA is still associated with cell wall at levels similar to that of for the full length AtlA.

### GelE cleaves AtlA near the terminus of Domain I between Ala 173 and Leu 174

**Fig 3:**
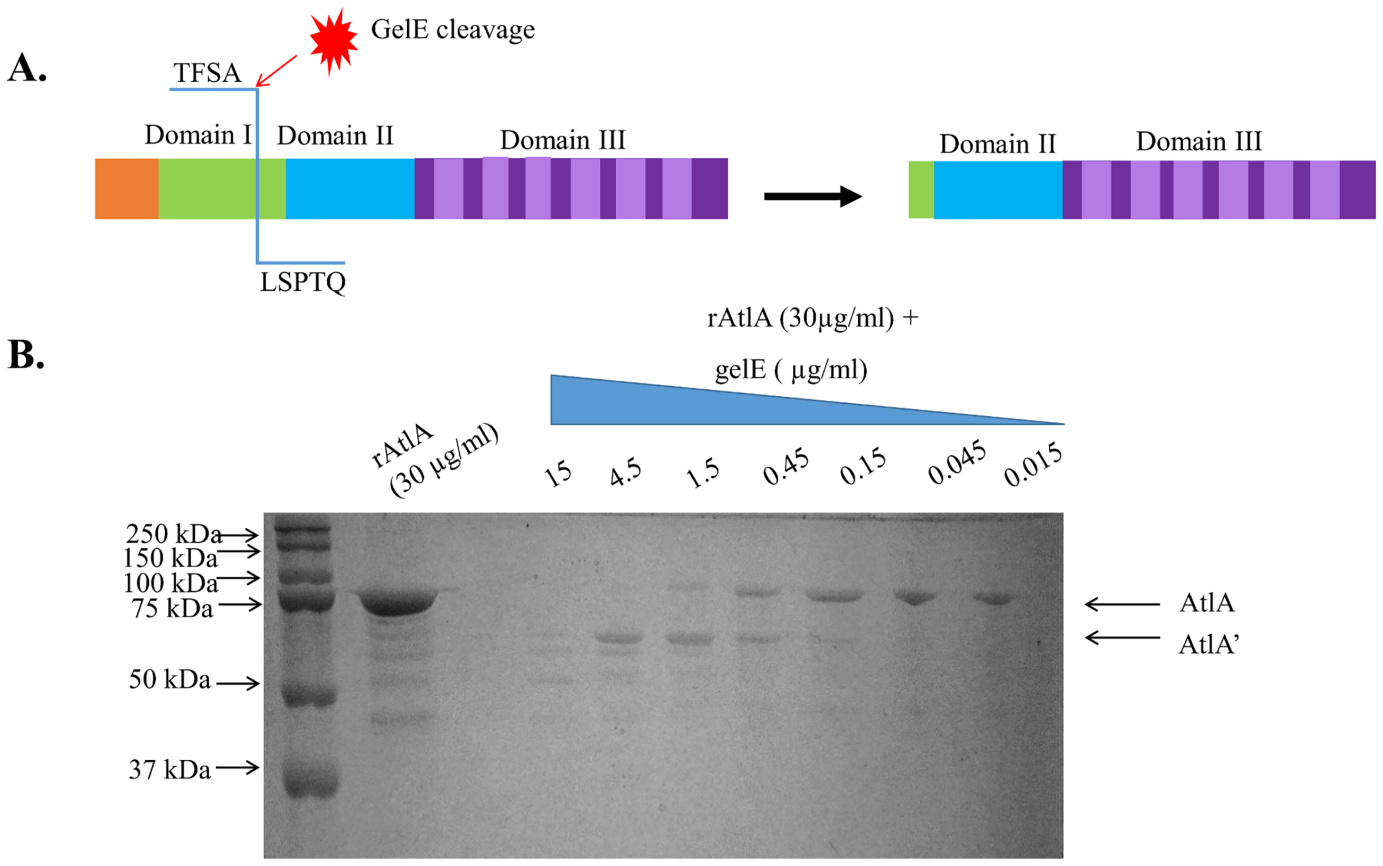
GelE cleaves AtlA between Ala 173 and Leu 174 within Domain I. (A) Truncated ~60 kDa band was N-terminally sequenced. The most prominent N-terminal sequence was identified as LSPTQ, suggesting that cleavage occurs between Ala 173 and Leu 174. (B) Cleavage of rAtlA by purified GelE. Sample lanes from right to left represent reaction mixtures containing 30 μg of rAtlA/ml with various amounts of purified GelE (0.015, 0.045, 0.15, 0.45. 1.5, 4.5, and 15 μg/ml), and rAtlA (30 μg/ml) alone. Molecular mass standards are indicated to the left in kilodaltons. Results presented in Fig 3B are representative of three independent experiments.

Our data thus far suggested that during GelE-mediated cleavage, a portion of AtlA remains on the surface of cells, and full-length and truncated versions of AtlA can be distinguished using domain specific mAbs. To determine the precise location of GelE-cleavage on recombinant AtlA an *in vitro* GelE cleavage assay was performed as shown in Fig 3B. N-terminal sequencing analysis on an isolated ~ 60 kDa AtlA band of recombinant AtlA (Fig 3B) cleaved by 10 μg/mL purified GelE for 30 minutes at 37°C identified the GelE-cleavage site as occuring between Ala 173 and Leu 174. This site is within Domain I and 7 amino acids from the N terminus of Domain II (Fig 3A). Based on this information, we constructed and purified an N-terminally truncated recombinant form of AtlA, rAtlA’, for further functional analysis. The molecular weight of this cleavage product is 58.9kDa, consistent with the band detected in Western blot analysis of GelE producing strains.

### Presence of GelE does not significantly change peptidoglycan hydrolase activity of AtlA

**Fig 4:**
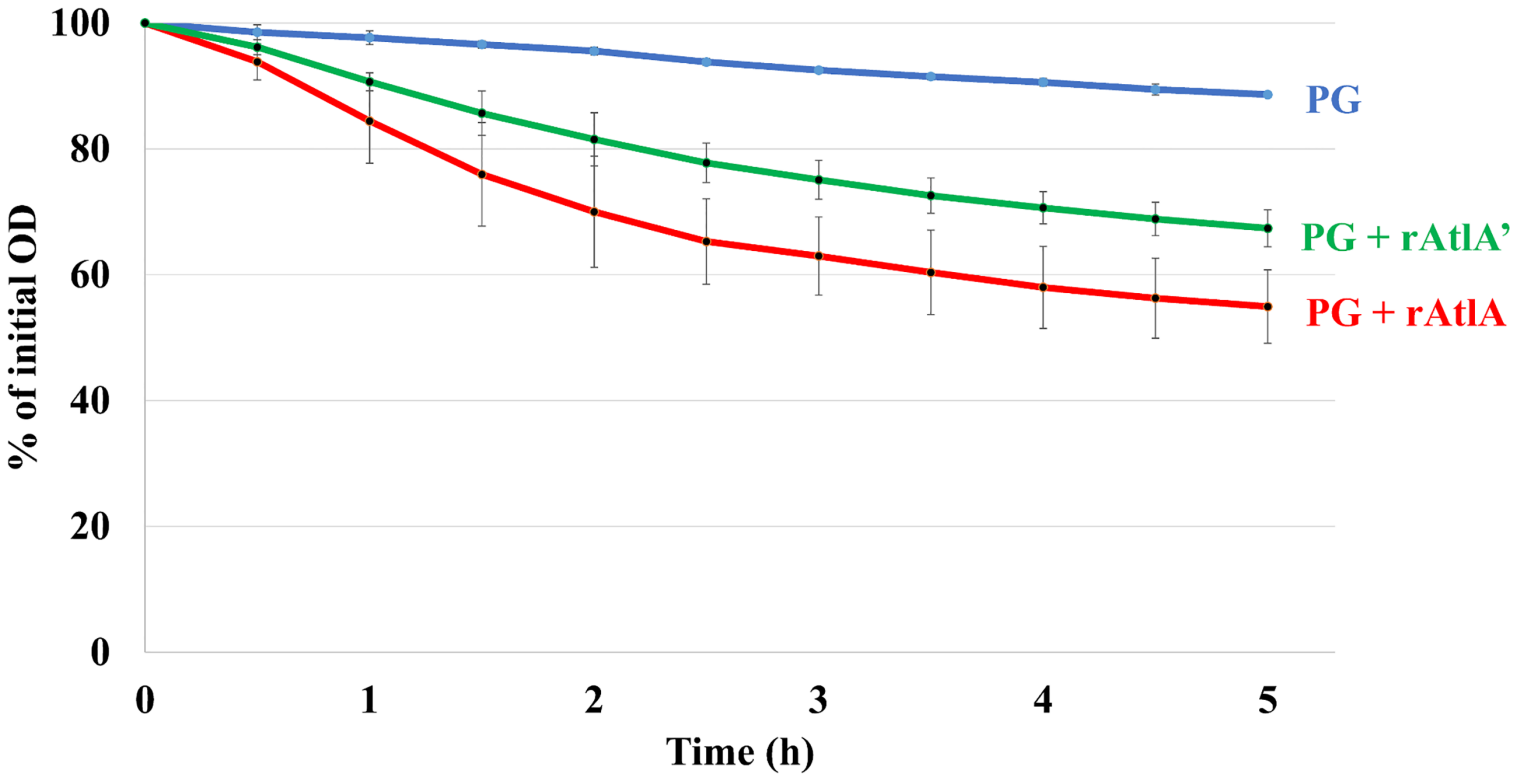
GelE-dependent AtlA cleavage does not significantly affect AtlA peptidoglycan hydrolysis activity. Equal molar concentrations (65 nM) of either recombinant full-length AtlA or AtlA’ were added to purified peptidoglycan and the peptidoglycan hydrolysis rate was measured by examining the of the solution every 30 minutes over a 5-hour time period. As a control, the hydrolysis rate for peptidoglycan alone was measured. Results are averages of three independent experiments. Error bars represent one standard deviation of the mean. Statistical significant differences (P < 0.05) in peptidoglycan hydrolysis were determined for PG + rAtlA and PG + rAtlA’ relative to PG only, using Student’s t test. No significant difference (P > 0.05) was determined for PG + rAtlA relative to PG + rAtlA’, using Student’s t test.

One of the major functions of AtlA is peptidoglycan cleavage [22]. To determine the effect GelE cleavage has on the ability of AtlA to cleave peptidoglycan, we used a peptidoglycan hydrolase assay to compare activities of rAtlA and rAtlA’ (Fig 4). As a control, peptidoglycan alone showed minimal peptidoglycan hydrolysis. The addition of either rAtlA or rAtlA’ showed a significant ability to hydrolyze peptidoglycan compared to the peptidoglycan only control, while the two forms of the enzyme demonstrated no significant difference in the ability to hydrolyze peptidoglycan (p < 0.05). This result demonstrated that GelE cleavage of AtlA does not significantly affect the peptidoglycan hydrolase activity of AtlA.

### GelE effects chain length partially through an AtlA interaction

It has been reported that both AtlA and GelE affect *E*. *faecalis* cell chain length [8,9,21]. We defined chain length as the number of cells per chain and categorized them as either a short, medium, long or extra-long chains as previously defined [41]. In three independent experiments 50 randomly chosen chains per strain were counted and the percentage of cells with less than or equal to 4 cells was determined (Table 1).

**Table 1:** Percentage of cells with four or fewer cells per chain.

	No treatment	+ rAtlA	+ rAtlA’
**OG1RF**	100% ± 0	N.D.	N.D.
**ΔatlA**	40% ± 5.3	98.7% ± 2.3	N.D.
**ΔatlAΔgelE**	28% ± 2	52.7% ± 3.8	93.3% ± 3.1

Averages of three independent experiments are shown in this table. ± indicates one standard deviations from the mean. All groups were compared to their no treatment control. *P*> 0.05 in all cases using Student’s *t* test.

**Fig 5:**
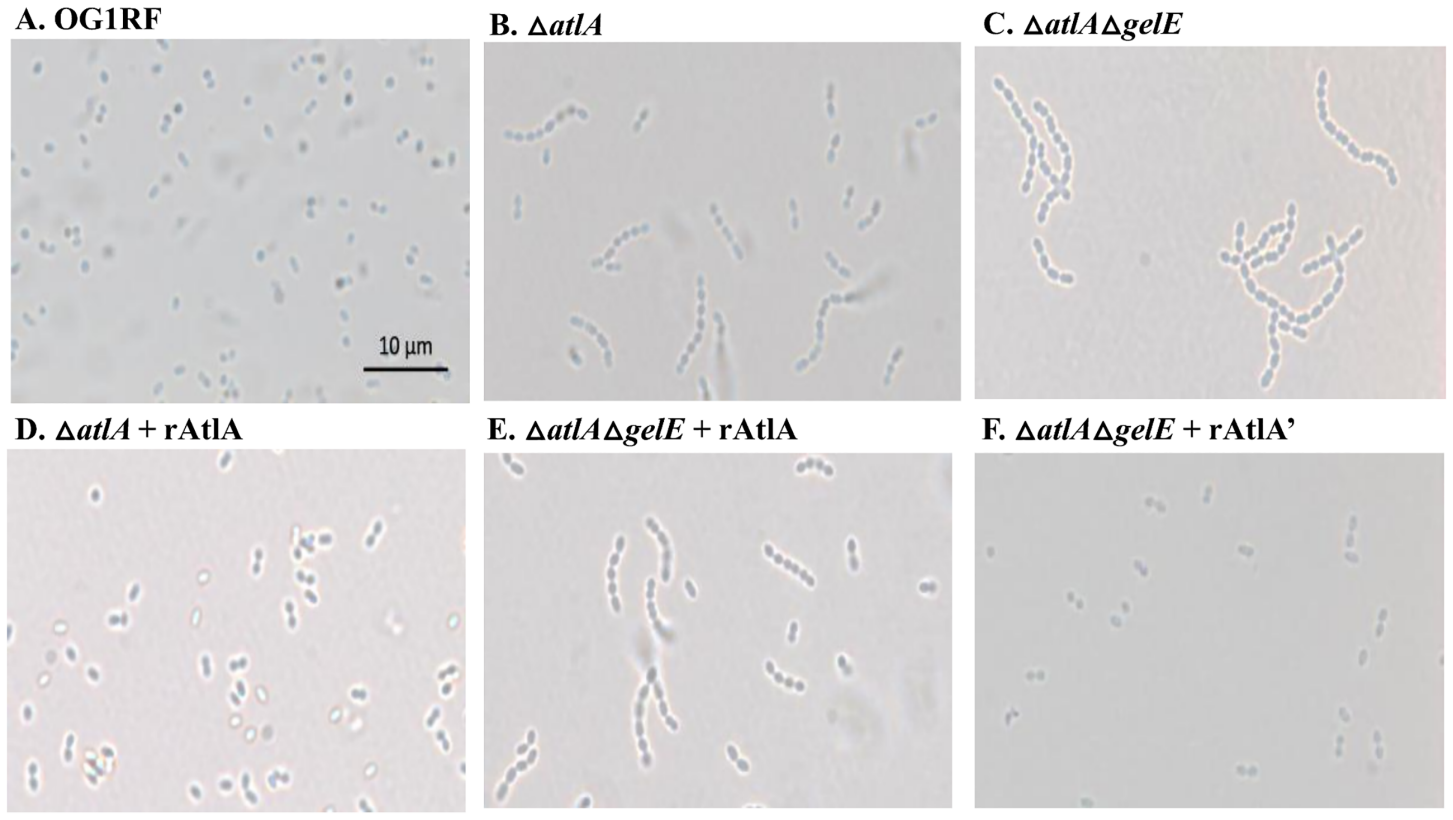
Presence of AtlA and GelE affects cellular chain length. OG1RF, OG1RFΔatlA, and OG1RFΔatlAΔgelE were harvested and imaged on a light microscope at 1000X magnification: (A) OG1RF (B) OG1RFΔatlA (C) OG1RFΔatlAΔgelE (D) OG1RFΔatlA + recombinant AtlA (E) OG1RFΔatlAΔgelE + recombinant AtlA (F) OG1RFΔatlAΔgelE + recombinant AtlA’. The scale bar in the lower right is 10 μm.

Consistent with results of Qin *et al*. [8], wild-type OG1RF cultured cells appeared in single or in pair forms, indicating separated cells (100% ± 0 are < 4 cells per chain) (Fig 5A), while OG1RF Δ*atlA* demonstrated a chain phenotype in which the cells were not properly separated and thus contained more than two cells in a chain (40% ± 5.3are < 4 cells per chain) (Fig 5B). Moreover, the absence of both AtlA and GelE (Δ*atlA*Δ*gelE*) resulted in a long chain phenotype (28% ± 2 are < 4 cells per chain) (Fig 5C). Given that a larger percentage of chained cells were observed in Δ*atlA*Δ*gelE* than the Δ*atlA* strain, suggests that GelE, in addition to AtlA modification, may affect chain length through other factors involved in septum cleavage.

We further examined whether the addition of recombinant AtlA could rescue the chaining phenotype observed in either the Δ*atlA* or Δ*atlA*Δ*gelE* strains. Upon the addition of recombinant AtlA to a Δ*atlA* strain, which expresses active GelE under our *in vitro* conditions, cells appeared as individual cells with short chains observed (98.7% ± 2.3 are < 4 cells per chain) (Fig 5D). The addition of full-length rAtlA to the Δ*atlA*Δ*gelE* strain displayed a medium chain phenotype (52.7% ± 3.9 are < 4 cells per chain) (Fig 5E) as opposed to the long chain observed in Fig 5C. However, the addition of recombinant AtlA’ to the Δ*atlA*Δ*gelE* strain restored the culture to the individual cell phenotype (93.3% ± 3.1 are < 4 cells per chain) (Fig 5F) similar to the wild-type phenotype observed in Fig 5A. Since the addition of rAtlA’, representing GelE-cleaved AtlA, restored the wild-type single or paired phenotype, this suggests that GelE-cleavage of AtlA promotes cell separation during division.

### GelE-dependent AtlA cleavage impacts AtlA localization to the cell septum and cell pole

**Fig 6:**
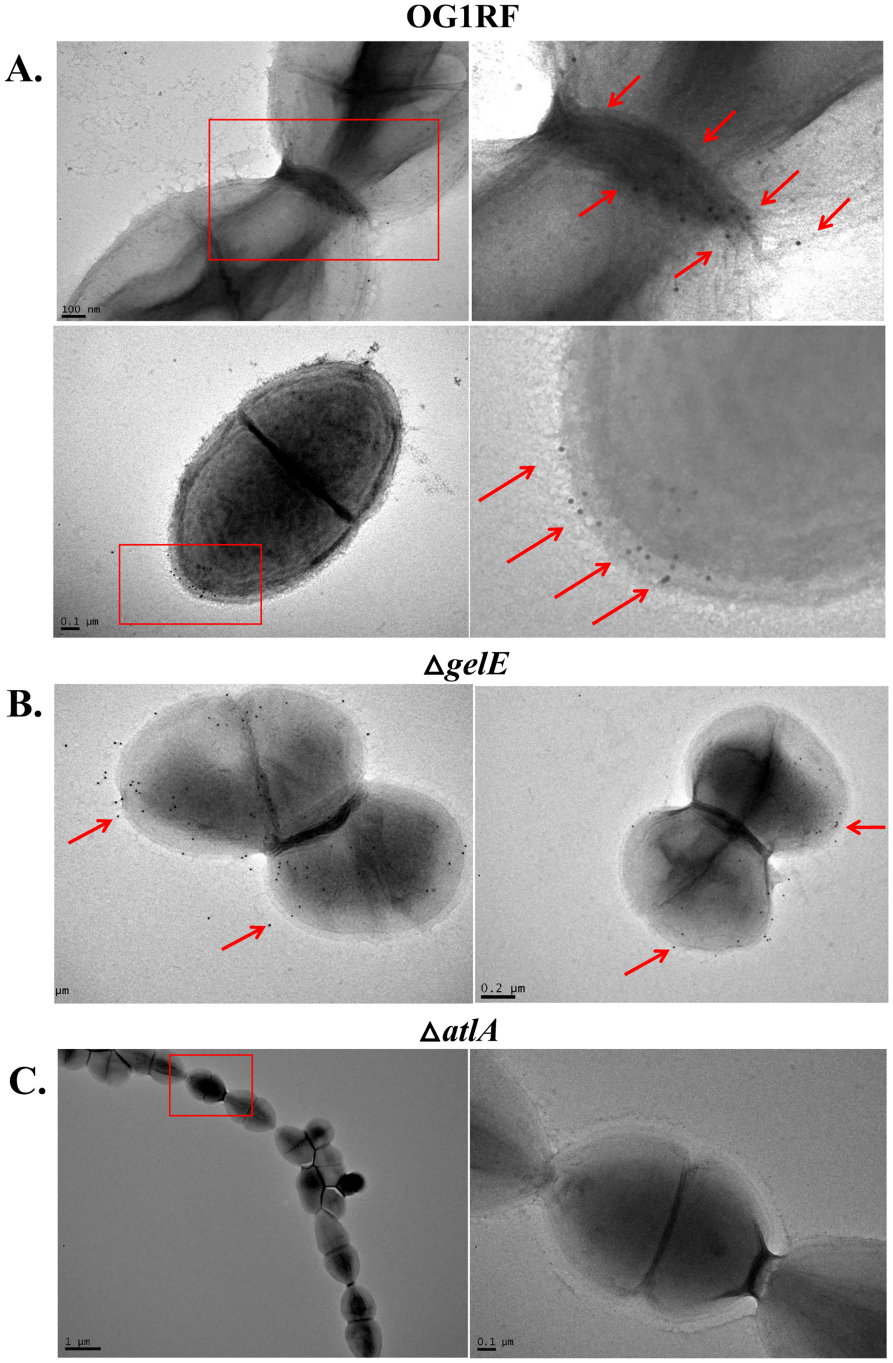
Impact of GelE activity on AtlA subcellular localization. OG1RF and *ΔgelE* were cultured overnight in BHI and placed on nickel-coated carbon grid. Anti-AtlA mAb 44 was used to label AtlA on the surface of cells. Samples were then labeled with gold-conjugated anti-mouse IgG and then imaged with a JEOL JEM-1400 electron microscope. (A) OG1RF (B) *ΔgelE*. (C) OG1RF and *ΔgelE E*. *faecalis* were equally divided into three regions by the distance to the center septum. For each strain, fifty cells at the late stages of cell division were selected for analysis.

Given that the presence of GelE-cleaved AtlA promoted daughter cell separation as visualized by light microscopy, we investigated whether GelE-mediated cleavage might affect AtlA localization on daughter cells prior to separation. As observed by electron microscopy, AtlA protein on the cell surface of wild-type OG1RF, as monitored by anti-AtlA mAb 44 which can detect full length AtlA and AtlA’, appeared to primarily localize to the cell septum region (Fig 6A). In a Δ*gelE* strain, using anti-AtlA mAb 44 to monitor AtlA localization, AtlA appeared dispersed across the surface of the cell rather than localizing to a specific area (Fig 6B). These observations are further confirmed by quantitative analysis of the site of AtlA localization on each bacterium. As shown in Fig 6C, majority of AtlA gold particles (73%) were localized in region 1, which is the equatorial region containing the cell division septum, comparing to 16% and 11% in regions 2 and 3, respectively. For the Δ*gelE* however, the distributions are 36%, 34%, and 30% for region 1, 2, and 3, respectively. Based on these results, we hypothesize that cell pole localization was dependent on the presence of GelE.

## Discussion

In *E*. *faecalis*, AtlA is the major peptidoglycan hydrolase. The ability to cleave peptidoglycan makes AtlA pivotal in separating dividing cells [8,9]. As such, AtlA requires strict modulation to regulate its function in cell division. Recent evidence suggests that multiple mechanisms control AtlA activity, including glycosylation [13]. The results presented here provide strong evidence that post-translational modification of AtlA by GelE directly affects the function of AtlA during cell division.

Under zymogram analysis, Eckert *et al*. demonstrated that AtlA displayed two protein bands that were present in the wild-type strain, but lacking in the OG1RF*ΔatlA* strain: one at 72 kDa (the approximate molecular weight of AtlA) and the other at 62 kDa [22]. Thomas *et al*. examined the impact proteases induced by the Fsr quorum sensing system of *E*. *faecalis* had on the autolytic profiles of wild type OG1RF, OG1RF*ΔgelE*, OG1RF*ΔgelEΔsprE*, and OG1RF*ΔsprE* cells. In surface autolysin profiles, a processed AtlA band of approximately 62kDa was observed only in cells producing GelE, whereas those lacking GelE (OG1RF*ΔgelE*, OG1RF*ΔgelEΔsprE)* expressed AtlA of approximately 72kDa [20]. Consistent with these results, our analysis of AtlA expression in different *E*. *faecalis* strains by Western blot showed that strains that expressed GelE (OG1RF and OG1RF*▽sprE*) displayed a major band consistent with the 62 kDa band observed while those strains that were incapable of producing GelE (OG1RF*ΔgelE* and OG1RF*ΔgelEΔsprE*) displayed a major band at approximately 72 kDa, the size of full length AtlA (Fig 1B). Together these results suggested that the presence of GelE is necessary for the processing of AtlA to the 62 kDa fragment. After further analysis, the GelE-cleavage site on AtlA was identified within Domain I. We showed that this cleavage site occurs between Ala 173 and Leu 174, near the start of the pre-defined Domain II (Fig 3). AtlA lacking this N-terminal region would have a molecular weight of 58.9 kDa, consistent with the approximate 62 kDa observed.

While work by Thomas *et al*. suggests a role for SprE-mediated processing of AtlA in regulating autolysis activity, [20] we were unable to find evidence supporting a major role of SprE processing of AtlA in mediating cell division, as GelE-processed AtlA alone could alleviate the chaining phenotype when incubated with our Δ*atlA*Δ*gelE* strain *in vitro*. In addition, analysis of different *E*. *faecalis* strains lacking SprE expression (OG1RF*▽sprE* and OG1RF*ΔgelEΔsprE*) had no detectable impact on AtlA bands detected by Western Blot analysis, producing results similar to that of OG1RF and OG1RF*ΔgelE* respectively. (Fig 1B) Taken together, these findings did not demonstrate an appreciable role for a SprE AtlA interaction in the regulation of cell division.

Once the GelE cleavage site on AtlA was identified, we sought to determine its impact on AtlA activity. Eckert *et al*. examined the enzymatic activity of different domain truncations of AtlA [22]. Truncation of Domain I did not significantly alter the enzymatic activity. In contrast, truncation of Domain III from Domain I and II led to a significant decrease in enzymatic activity, suggesting that Domain III is required for enzymatic activity [22]. Domain III contains six LysM molecules that confer AtlA the ability to bind to peptidoglycan. Based on these findings, Eckert *et al*. proposed that Domain III is required to bring Domain II within the vicinity of its peptidoglycan substrate [22]. Consistent with our work, cleavage of the N-terminal domain by GelE did not significantly impact the ability of AtlA to hydrolyze peptidoglycan (Fig 4).

OG1RF*ΔatlA* grow as long chains linked together at their cell septa, unable to properly separate [8,9]. Similarly, OG1RF*ΔgelE* cells also display a chaining phenotype, suggesting that GelE is required for cell separation. Upon the complementation of a OG1RF*ΔgelE* strain with *gelE*, the cells appeared either in pairs or as single cells, further suggesting that the presence of GelE impacts cell division [21]. To determine the impact of GelE on chain length, Arias and Murray evaluated different *E*. *faecalis* clinical isolates for the expression of GelE and degree of chaining. These findings demonstrated that the absence of GelE did not necessarily correlate with a long chain phenotype [41]. However, if GelE must act though AtlA to mediate cell separation as our results suggest, any disruption of AtlA, regardless of the GelE expression, would present as a long chain phenotype and explain the lack of correlation seen between GelE expression and chaining. Alternatively, other mechanisms may be involved in *E*. *faecalis* cell separation independent of GelE, as suggested by Salamaga *et al*. [13].

During early growth stages long chains of cells are produced [44]. In *Streptococcus pneumoniae*, a streptococcus that has both short and long chain phenotypes similar to *E*. *faecalis*, the long chain phenotype is associated with an advantage in human epithelial cell adherence due to an increase in the number of adhesive events per particle [11]. Moreover, it was hypothesized that a short chain length in *S*. *pneumoniae* allows the bacterium to evade host immune defense cells, such as phagocytes, promoting invasive disease [45]. Based on these findings, we speculate that human cell adherence would be most important at early stages of growth, potentially prior to Fsr signaling, when GelE cleavage of AtlA has not yet occurred and long cell chains are observed. As GelE reaches maximum activity levels at later growth stages, after colonization is established, AtlA would be cleaved and short cell chains would result. These short chains could potentially aid in bacterial dissemination or help *E*. *faecalis* evade host immune defense. GelE has previously been shown to be associated with an increase in bacterial burden at dissemination sites [46]. Thus, GelE could aid in bacterial cell dissemination by cleaving AtlA at a later growth stage, resulting in short chains. The short chained *E*. *faecalis* cellshave been shown to have decreased phagocytosis in a zebrafish infection model [13], further suggesting that the GelE processing of AtlA may impact *E*. *faecalis* virulence.

Using our anti-AtlA mAb to track both GelE-cleaved and full-length AtlA, localization of AtlA in wild-type OG1RF and OG1RF*ΔgelE* cells was monitored via electron microscopy. Wild-type OG1RF cells labeled with anti-AtlA mAb 44 showed localization of AtlA to the cell septum (Fig 6A) while OG1RF*ΔgelE* cells labeled with anti-AtlA mAb 44 showed random localization of AtlA across the cell surface (Fig 6B). These observations indicate that the presence of GelE is required for AtlA to accumulate at the cell septum.

While our evidence independently demonstrates that GelE-mediated cleavage of AtlA is sufficient to encourage cell separation and cleaved AtlA localization to the cell septum, our efforts to date have not yet provided a complete understanding of the mechanism which mediates these events. In staphylococci, the major autolysin, AtlA, was found to localize to the cell septum due to an interaction with one of the major peptidoglycan constituents, lipoteichoic acid (LTA) [47], while wall teichoic acid (WTA), which is lower in abundance at the staphylococcal cell septum, is thought to prevent AtlA binding [48]. Based on these results, future work will explore the role of teichoic acid on the localization of cleaved AtlA. Continued study of will provide a deeper understanding of cell separation during the final stage of cell division and the potential importance of coordinating cell separation and cellular density through Fsr mediated GelE expression.
